# Subjektive Erfahrungen in der Anpassung an die Pension: Qualitative Befunde aus der Schweiz

**DOI:** 10.1007/s00391-023-02190-3

**Published:** 2023-05-09

**Authors:** Julia Reiner, Sabina Misoch

**Affiliations:** Institut für Altersforschung (IAF), OST – Ostschweizer Fachhochschule, Rosenbergstr. 59, 9001 St. Gallen, Schweiz

**Keywords:** Übergang in die Pension, Anpassungsprozess, Qualitative Interviews, Lebenszufriedenheit, Ressourcen, Transition to retirement, Adjustment process, Qualitative interviews, Life satisfaction, Resources

## Abstract

**Hintergrund:**

In der bisherigen Forschung zur Anpassung an die Pension dominieren quantitative Studien zur Entwicklung verschiedener Indikatoren des Anpassungserfolgs. Demgegenüber mangelt es an qualitativen Untersuchungen, welche die subjektiven Erfahrungswerte der Betroffenen im Anpassungsprozess an die Pension betrachten.

**Fragestellung:**

Der Beitrag behandelt die subjektiven Anpassungserfahrungen von Personen, deren regulärer Pensionseintritt rund ein Jahr zurückliegt. Im Fokus stehen erlebte Gewöhnungserfordernisse und subjektiv relevante Faktoren der Lebenszufriedenheit.

**Material und Methoden:**

Die Befunde basieren auf qualitativen Interviewdaten, die im Rahmen einer umfassenderen, zwischen 2019 und 2022 in der Deutschschweiz durchgeführten Mixed-Methods-Studie gewonnen wurden. Im Zuge dessen erfolgten 25 leitfadengestützte Interviews, die inhaltsanalytisch ausgewertet wurden.

**Ergebnisse:**

Der Pensionseintritt wurde mehrheitlich positiv erlebt, was v. a. mit einem Freiheitsgewinn und mit Regenerationsmöglichkeiten begründet wurde. Die Zeit nach der Pensionierung barg jedoch auch Gewöhnungserfordernisse in unterschiedlichen Lebensbereichen. Die Lebenszufriedenheit stieg gegenüber der Erstbefragung vor der Pensionierung an und wurde mit verschiedenen zu- und abträglichen Faktoren in Verbindung gebracht, die von persönlichen Merkmalen bis hin zu gesellschaftlichen Entwicklungen reichten.

**Diskussion:**

Die Pensionierung stellt keine zwangsläufig krisenhafte Erfahrung dar, kann jedoch von anderen kritischen Ereignissen begleitet werden. Ein zufriedenstellendes Leben in der Pension steht mit multiplen Faktoren in Verbindung, unter denen die individuelle Ressourcenausstattung eine wesentliche Rolle spielt.

## Abstract (en)

**Background:**

Previous research on retirement adjustment has been dominated by quantitative studies on the development of different indicators for adjustment success. In contrast, there is a lack of qualitative studies examining the subjective experiences of retirees in their adjustment process to the new life phase.

**Objective:**

The article deals with the subjective adjustment experiences of persons whose regular retirement was about 1 year ago. It focuses on experienced habituation requirements and subjectively relevant factors of life satisfaction.

**Material and methods:**

The findings are based on qualitative interview data from a more comprehensive mixed methods study conducted between 2019 and 2022 in the German-speaking part of Switzerland. In the course of the study, 25 semi-structured interviews were carried out and analyzed using qualitative content analysis.

**Results:**

Entering the new life phase was predominately experienced positively, which was mainly associated with a gain in freedom and opportunities for regeneration; however, the time after retirement also entailed habituation requirements in different areas of life. Life satisfaction increased compared to the initial survey before retirement and was associated with various beneficial and critical factors, ranging from personal characteristics to social developments.

**Conclusion:**

Retirement itself is not necessarily a critical experience but can be accompanied by other critical events. A satisfactory life 1 year after retirement is linked to multiple factors. Among these, resources play an essential role.

Im Zuge des demografischen Wandels erhält die Frage nach der Anpassung an die Pension verstärkt Gewicht. In der einschlägigen Forschungsliteratur dominieren quantitative Studien zur Entwicklung von Indikatoren für den „Anpassungserfolg“ der Betroffenen, während vergleichsweise wenige Beiträge zur qualitativen Exploration subjektiver Anpassungserfahrungen vorliegen. Vor diesem Hintergrund wurde auf Basis von 25 qualitativen Interviews untersucht, wie pensionierte Personen das erste Jahr nach dem Übergang in die neue Lebensphase erleben.

## Hintergrund und Fragestellung

Mit dem demografischen Wandel [5] stellen sich nicht nur neue gesamtgesellschaftliche Fragen, sondern auch solche der individuellen Pensionsgestaltung auf der Ebene der Betroffenen. Inzwischen handelt es sich bei der Pension um einen ausgedehnten Lebensabschnitt, der sich gegenüber früheren Dekaden nicht nur durch durchschnittlich mehr Lebensjahre, sondern auch durch mehr Jahre in guter Gesundheit auszeichnet [6]. Diese und weitere Ausgangsbedingungen gegenwärtiger Pensionierungskohorten gehen mit neuen Gestaltungsmöglichkeiten und -anforderungen in Bezug auf diese Lebensphase einher [11].

War die Betrachtung der Pensionierung im frühen Fachdiskurs noch primär von einer defizitorientierten Perspektive geprägt, dominiert inzwischen das Verständnis eines Lebensübergangs mit Gewinnen und Verlusten, der mit einem entsprechenden, nicht notwendigerweise krisenhaften, Anpassungsbedarf einhergeht [13, 18]. Neben anderen Schwerpunkten bildet die Anpassung an die Pension einen nach wie vor großen Bereich der Pensionierungsforschung [22]. Konzeptuell wird dabei von einem mehrdimensionalen Prozess ausgegangen, der unterschiedliche Lebensbereiche betrifft und sich, einschließlich der Übergangserfahrung selbst, über mehr oder weniger lange Zeit in der Nacherwerbsphase erstreckt [21]. Neben dem Umgang mit dem Verlust der Arbeit kann die Entwicklung eines zufriedenstellenden Lebensstils als ein wesentliches Anpassungserfordernis in der neuen Lebensphase erachtet werden [19]. In der bisherigen Beschäftigung mit der Anpassung an die Pension dominieren drei theoretische Zugänge [18, 21]: (a) rollentheoretische Ansätze, (b) die Kontinuitätstheorie von Atchley [2] sowie (c) die Lebenslaufperspektive [9]. Letztere wird als vergleichsweise umfassendere Perspektive auf die Anpassung an die Pension bewertet, die sowohl die Betroffenen als handelnde Akteur:innen als auch die kontextuelle Einbettung der Übergangserfahrung berücksichtigt [20].

In der bisherigen Pensionierungsforschung wurde die Anpassung an die Pension überwiegend in quantitativen Studien mit Blick auf die Entwicklung verschiedener, oftmals das Wohlbefinden betreffende Indikator-Variablen untersucht [18]. Obwohl dieser Zugang wertvolle Einblicke in die Entwicklung der jeweiligen Indikatoren und diesbezügliche interindividuelle Unterschiede lieferte, sind damit auch Limitationen verbunden. Diese betreffen etwa die Fokussierung auf das Outcome der Pensionierungs- bzw. Anpassungserfahrungen zu einem jeweiligen Zeitpunkt, während Letztere selbst unterbelichtet bleiben [12, 18, 19]. Insofern werden auch mehr Beiträge zu den subjektiven Erfahrungen in der Anpassung an die neue Lebensphase aus dem Alltag der Betroffenen gefordert [1, 10], für deren offene Exploration sich gerade qualitative Ansätze eignen. Diesbezügliche Einblicke scheinen nicht zuletzt für ein tiefergehendes Verständnis für den Anpassungsprozess selbst, über die Fokussierung auf dessen „Ergebnis“ hinaus, von Bedeutung.

Vor diesem Hintergrund beschäftigt sich der vorliegende Beitrag mit den subjektiven Erfahrungen in der Anpassung an die Pension aus Sicht von Personen, welche sich seit rund einem Jahr in der Pension befinden. Im Zuge dessen sollen Einblicke in das Erleben der unmittelbaren Zeit nach der Pensionierung, erfahrene Gewöhnungserfordernisse und subjektiv relevante Faktoren für die Lebenszufriedenheit in der Pension herausgearbeitet werden.

## Studiendesign und Untersuchungsmethoden

Die nachstehenden Befunde wurden im Zuge eines umfassenderen Projekts zum Thema „Identitätskonstruktionen für den Ruhestand“ gewonnen, welches von 2019 bis 2022 durchgeführt und vom Schweizerischen Nationalfonds (SNF) finanziert wurde. Das Design umfasste zwei Erhebungszeitpunkte, bestehend aus einer standardisierten Erstbefragung von *n* = 400 erwerbstätigen, in der Deutschschweiz lebenden Personen, die sich rund ein halbes Jahr vor ihrem regulären Pensionsalter (Frauen: 64 Jahre, Männer: 65 Jahre) befinden.^1^ Zu den Ausschlusskriterien zählte u. a. die Absicht, den Bezug der Altersrente aufzuschieben und in der Pension in Vollzeit weiterzuarbeiten. Neben dem gesetzlichen Pensionsalter orientierte sich die Definition des Pensionierungszeitpunkts demnach am Rentenbezug und an der Unterschreitung eines Vollzeitbeschäftigungsausmaßes – Kriterien, die neben anderen zu gängigen Bestimmungsfaktoren des Status als pensionierte Person zählen [7]. Im Unterschied zum Kriterium des gänzlichen Arbeitsmarktaustritts bot diese Vorgehensweise den Vorteil, auch solche Personen in die Untersuchung einzuschließen, die eine Teilzeitbeschäftigung als Teil ihres Lebensentwurfs für die Pension erachten oder einer solchen aus finanziellen Gründen nachgehen müssen.

Rund eineinhalb Jahre später erfolgten leitfadengestützte Interviews [16] mit *n* = 25 Teilnehmer:innen der Erstbefragung. Mit der Wahl dieses Nachbefragungszeitpunkts sollte auf die Erfahrungen unmittelbar nach der Pensionierung fokussiert werden, ohne Anspruch auf die Abbildung mittel- und längerfristiger Entwicklungen, wofür längere Erhebungszeiträume notwendig wären. Insofern versteht sich der gewählte Nachbefragungszeitpunkt nur als „Momentaufnahme“ im Rahmen eines länger andauernden Anpassungsprozesses an die Pension. Die Selektion der Interviewpartner:innen erfolgte nach maximalkontrastierten Merkmalen [17], zu welchen u. a. soziodemografische Charakteristika und solche der Ressourcenausstattung zählten. Die Auswertung erfolgte mittels qualitativer Inhaltsanalyse [14] und umfasste deduktive und induktive Kategorienbildungen.

## Ergebnisse

### Sample

Bei den Interviewpartner:innen handelte es sich zu annähernd ausgeglichenen Anteilen um Frauen (48,0 %) wie Männer (52,0 %). Die Mehrheit bildeten Personen mit einer Schweizer Nationalität (96,0 %). Beim Bildungsniveau zeigte sich ein Überwiegen von Personen mit einem Abschluss auf Tertiärstufe (60,0 %) gegenüber Befragten mit einer Ausbildung bis Sekundarstufe II (40,0 %). Mit 60,0 % waren die meisten Interviewpartner:innen verheiratet oder in einer Partnerschaft, während 40,0 % alleinstehend waren. Zum Interviewzeitpunkt waren 60,0 % der Befragten nicht mehr beschäftigt. Die übrigen Personen gingen seit ihrer Pensionierung in gleicher oder veränderter Weise einer Teilzeitbeschäftigung nach.

### Die unmittelbare Zeit nach der Pensionierung

Die Zeit nach dem Pensionseintritt wurde mehrheitlich positiv erlebt und teilweise mit einem urlaubsähnlichen Gefühl in Verbindung gebracht, welches einzelne Wochen bis Monate andauerte. Das positive Erleben dieser Zeit wurde vielfach in einem neuen Freiheitsgefühl und veränderten Selbstbestimmungsmöglichkeiten begründet. Mehrere Befragte hoben auch die Möglichkeit zur Regeneration von akuten Erschöpfungszuständen und psychischen Belastungen am Arbeitsplatz als positive Erfahrung hervor.

Sofern unmittelbar nach dem Übergang in die Pension Belastungen erfahren wurden, standen diese in Verbindung mit kritischen Ereignissen wie Sterbefällen im sozialen Umfeld, Gesundheitsproblemen oder der Nicht-Realisierung ursprünglicher Pläne, beispielsweise infolge der COVID-19-Pandemie. Abgesehen davon fühlten sich einzelne Personen nach ihrem Pensionseintritt durch aufgekommene oder unveränderte subjektive Leistungsansprüche belastet. Diese bezogen sich auf die berufliche Tätigkeit oder auch auf die Haushaltsführung.

### Subjektive Gewöhnungserfordernisse

Die Befragten berichteten für die Zeit nach der Pensionierung verschiedene und teilweise als herausfordernd erlebte Gewöhnungsprozesse. Diese betrafen zunächst gesundheitliche Probleme oder als altersbedingt eingestufte Veränderungen auf körperlicher und kognitiver Ebene. Letztere wurden von einigen Betroffenen bereits seit mehreren Jahren an sich beobachtet, rückten jedoch aufgrund der Pensionierung und deren Bewertung als Schritt in die letzte Lebensphase oder aus anderen Gründen (wie Sterbefällen im sozialen Umfeld) in der Pension verstärkt ins Bewusstsein.

Finanzielle Veränderungen bildeten einen weiteren Gegenstand berichteter Gewöhnungsprozesse. Mussten sich einzelne Befragte an die verringerten finanziellen Spielräume gegenüber der Zeit vor der Pensionierung erst gewöhnen, bildeten diese für mehrere andere unter Verweis auf ihre finanziellen Vorbereitungen keine Überraschung. Mehr als in der Veränderung des monatlichen Einkommens bestand ein Gewöhnungserfordernis für einige Personen darin, staatliche Gelder zu erhalten oder generell Geld zu erhalten, ohne eine Leistung dafür zu erbringen.

Thematisiert wurde auch der Verlust beruflicher Kontakte und mit ihm die gewöhnungsbedürftige Vorstellung, sich in der Pension aktiv um die Pflege sozialer Kontakte bemühen zu müssen, während diese vor der Pensionierung noch ein selbstverständliches Element des Berufsalltags waren. Andere Themen speziell unter erwerbstätigen Befragten bildeten das Senken individueller Ansprüche an die Ausübung der beruflichen Tätigkeit und die permanente Erreichbarkeit für berufliche Kontakte. Andere Personen verwiesen auf die Entschleunigung ihres Alltags oder das Fehlen von Reisetätigkeiten als festen Baustein des früheren Berufsalltags, womit sie sich in der Pension erst noch zurechtfinden mussten.

### Subjektiv relevante Faktoren der Lebenszufriedenheit

Die allgemeine Lebenszufriedenheit wurde neben der Erstbefragung auch in der qualitativen Nachbefragung zunächst in Form eines Single-Item [4] erfasst. Die durchschnittliche Lebenszufriedenheit der Interviewpartner:innen fällt zum Nachbefragungszeitpunkt in den obersten Wertebereich (M = 8,2, SD = ±1,1; möglicher Range: 0–10). Im Verhältnis zum Erstbefragungszeitpunkt (M = 7,5, SD = ±1,7) zeigte sich unter Anwendung des Wilcoxon-Tests für abhängige Stichproben eine signifikante Zunahme der allgemeinen Lebenszufriedenheit (*z* = −2336, *p* = 0,019).

In den Interviews wurden zu- und abträgliche Faktoren der Lebenszufriedenheit aus Sicht der Befragten exploriert. Soziale Ressourcen zählten zu den am häufigsten thematisierten förderlichen Faktoren, wobei v. a. der zentrale Stellenwert familiärer Beziehungen hervorgehoben wurde. Zwei weitere, mehrfach genannte Bereiche bildeten finanzielle und gesundheitliche Ressourcen. Der Wert einer soliden finanziellen Situation wurde primär in ihrem Beitrag für das subjektive Absicherungsgefühl verortet sowie darin, sich persönlich wichtige Dinge (auch von nicht-materiellem Wert) leisten zu können. Beschwerdefreiheit und eine insgesamt gute Gesundheit wurden wiederum als zentrale Voraussetzung für die Breite an Gestaltungsoptionen für die Pension bewertet und in dieser Relevanzsetzung auf die Lebenszufriedenheit bezogen.

Neben verschiedenen Dimensionen der Ressourcenausstattung nannten die Befragten noch weitere förderliche Faktoren für die Zufriedenheit mit ihrem Leben. Hierzu zählten persönliche und biografische Merkmale wie ein gutes Verhältnis zu sich selbst, der Rückblick auf ein erfülltes Leben sowie die abgeschlossene Aufarbeitung persönlicher Lebensthemen. Ebenso viele Befragte thematisierten Aspekte ihrer gegenwärtigen Lebensführung, beispielsweise die Pflege eines achtsamen und gegenwartsorientierten Lebensstils, das Fehlen von Langeweile sowie das Verfolgen persönlicher Projekte.

Weitere Faktoren, die als zuträglich für die Lebenszufriedenheit erachtet wurden, betrafen die individuellen Wohnbedingungen, das Fehlen von Problemen und kritischen Ereignissen sowie die Erfahrung von Freiheit und Selbstbestimmung. Im letzteren Fall sind es v. a. die mit der Pensionierung erlebten zeitlichen Veränderungen in Form von mehr Zeitbudget oder -hoheit, die in Beziehung zum subjektiven Freiheitsgefühl gesetzt wurden. Mehrfach wurde in diesem Zusammenhang jedoch auch die Relevanz von Ressourcen für die Nutzung der neuen Freiheit thematisiert. Einzelne Befragte führten schließlich die Möglichkeit zur Weiterführung ihrer beruflichen Tätigkeit und ihre frühen Vorbereitungen auf die Pension als förderliche Faktoren für ihre Lebenszufriedenheit an.

Auch der Lebenszufriedenheit abträgliche Faktoren waren Gegenstand der Interviews. Rund ein Fünftel der Befragten führte keine spezifischen Faktoren an; stattdessen wurde in diesen Fällen die Ansicht vertreten, dass man generell nicht vollends zufrieden sein kann und es im Alltag immer etwas gibt, woran man sich stört. Demgegenüber nannten ebenso viele Personen die COVID-19-Pandemie und mit ihr verbundene Einschränkungen im Alltag oder in der Planbarkeit von Vorhaben. Weitere Nennungen bezogen sich auf die Lebensgestaltung in der Pension, beispielsweise indem persönliche Pläne (aus anderen Gründen als der COVID-19-Pandemie) noch nicht realisiert werden konnten oder es zum Befragungszeitpunkt noch an Gestaltungsideen für die neue Lebensphase fehlte.

Erneut wurde unter den Befragten die Bedeutung von Ressourcen thematisiert: So wurden gesundheitliche Beeinträchtigungen mit z. T. deutlichen Abstrichen der Lebenszufriedenheit in Verbindung gebracht; dies gilt auch für bescheidene finanzielle Spielräume und Belastungen im Bereich sozialer Beziehungen. Darüber hinaus wurde auf gesamtgesellschaftliche Entwicklungen wie den Klimawandel oder Fragen sozialer Ungleichheit verwiesen. Im Einzelfall bestand ein abträglicher Faktor der Lebenszufriedenheit in bestimmten persönlichen Merkmalen, zu denen u. a. als schwer veränderbar wahrgenommene „Ticks“ gezählt wurden.

## Diskussion

Die erste Zeit nach der Pensionierung wurde von den meisten Interviewpartner:innen positiv erlebt, was insbesondere mit einem neuen Freiheitsgefühl oder Erholungsmöglichkeiten in Verbindung gebracht wurde. Diese und weitere Ergebnisse zur Entwicklung der Lebenszufriedenheit gegenüber der Zeit vor der Pensionierung können zunächst als stützender Befund für die Kontinuitätsannahme [2] bewertet werden, welcher von einem geringen Krisenpotenzial der Pensionierung ausgeht und auch in vergangenen Studien bereits mehrfache empirische Bestätigung erfahren hat [10]. Allerdings zeigen die Ergebnisse auch, dass der Übergang in die Pension mit anderen einschneidenden Ereignissen und damit verbundenen Belastungserfahrungen zeitlich zusammenfallen kann. Dies spricht für die notwendige Berücksichtigung von kritischen und teils unerwarteten Ereignissen für das Verständnis von Pensionierungsverläufen und interindividuellen Unterschieden in der Pensionierungs- bzw. Anpassungserfahrung. Darin kommt nicht zuletzt der Mehrwert der Lebenslaufperspektive als theoretischer Zugang zur Pensionierung zum Ausdruck, die mit ihren Prinzipien wie „linked lives“ und „time and place“ für die Beachtung entsprechender Kontextfaktoren (z. B. Sterbefälle, COVID-19-Pandemie) sensibilisiert [8]. Nachdem sich solche Ereignisse, auch in den vorliegenden Daten, teilweise als Veränderung der Ressourcenausstattung äußern (etwa gesundheitlicher oder sozialer Art), zeigt sich damit auch die Relevanz der vergleichsweise neueren „resource-based dynamic perspective“ [20], welche die Beachtung der Ressourcen*entwicklung* für das Verständnis der Anpassung an die Pension hervorhebt. Mit der Verschiebung des Blickwinkels auf die Mechanismen, durch welche die Pensionierungserfahrung das Wohlbefinden der Betroffenen beeinflussen kann, beansprucht diese Perspektive, über bestehende theoretische Zugänge hinauszugehen und zur Weiterentwicklung der theoretischen Auseinandersetzung mit der Anpassung an die Pension beizutragen [20]. Die Autor:innen sensibilisieren dabei auch für verschiedene Faktoren, welche die individuelle Ressourcenentwicklung beeinflussen können und von individuellen Merkmalen bis hin zu Faktoren auf der Makroebene reichen. Diese umfassende Betrachtungsweise trägt der Dynamik verschiedener Arten von Ressourcen Rechnung, für deren Verständnis sowohl subjektseitige als auch kontextuelle Faktoren zu bedenken sind, beispielsweise im Falle sozialer Netzwerke.

In den Ergebnissen wurde sichtbar, dass eine mehrheitlich positive Erfahrung der ersten Zeit nach der Pensionierung und eine insgesamt hohe Lebenszufriedenheit nach dem ersten Pensionsjahr nicht mit dem Fehlen von Gewöhnungserfordernissen in der neuen Lebensphase gleichzusetzen ist. Dies spricht für die Notwendigkeit einer Differenzierung zwischen dem Anpassungsprozess selbst und dessen Ergebnis im Sinne des individuellen Anpassungserfolgs. So können beide unterschiedlichen Erfahrungswerten unterliegen, beispielsweise indem einer gelungenen Anpassung ein herausfordernder Anpassungsprozess in bestimmten Lebensbereichen vorausgegangen sein kann [18]. Dies indizieren auch manche Erfahrungen im vorliegenden Sample, nachdem die berichteten Gewöhnungsprozesse teilweise als herausfordernd erlebt wurden, auch wenn die Lebenszufriedenheit nach dem ersten Pensionsjahr hoch ausfiel.

Ausgehend von den subjektiven Einschätzungen der Befragten unterliegt die Lebenszufriedenheit vielfältigen Faktoren, die von persönlichen Merkmalen bis hin zu gesamtgesellschaftlichen Entwicklungen reichen. Somit kommt auch in den subjektiven Relevanzsetzungen der Betroffenen die Mehrdimensionalität an Faktoren zum Ausdruck, die in Beziehung zur Anpassung an die Pension zu bedenken sind. Hierfür sensibilisiert etwa auch die Lebenslaufperspektive [9] ebenso wie die bisherige Forschungsliteratur zu Prädiktoren für die Anpassung an die Pension [3, 18]. Wie die Einschätzungen der Befragten nahelegen, spielt die individuelle Ressourcenausstattung für ein zufriedenstellendes Leben in der Pension eine wichtige Rolle, das insofern auch als wesentliche Frage von sozialen und gesundheitlichen Ungleichheitslagen erscheint. Arbeits- und pensionierungsspezifische Umstände fanden in der Bewertung der Lebenszufriedenheit ein Jahr nach der Pensionierung hingegen kaum Erwähnung. Allerdings prägten ursprüngliche Arbeitsbedingungen z. T. die Erfahrung der unmittelbaren Zeit nach der Pensionierung, in der für manche Befragte die Regeneration von arbeitsbezogenen Belastungen im Vordergrund stand.

## Limitationen

Für die Bewertung der Ergebnisreichweite sind verschiedene Limitationen zu bedenken, die u. a. in der Zusammensetzung der Interviewpartner:innen bestehen: Trotz maximal kontrastierender Fallauswahl konnten nur einzelne Personen rekrutiert werden, die ein geringes Bildungsniveau (im Sinne eines obligatorischen Schulabschlusses) aufwiesen und sich zum Erstbefragungszeitpunkt in der untersten Einkommenskategorie befanden. Aufgrund dessen und angesichts der erzielten Befunde erscheinen weitere qualitative Studien zu den subjektiven Anpassungserfahrungen speziell von Personen in prekären Lebenslagen als empfehlenswert. Darüber hinaus könnte in künftigen Studien stärker auf die Bedeutung einzelner Ressourcen für die Anpassung an die Pension fokussiert und diese bereits im Rahmen der Erhebung differenzierter exploriert werden. Neben der Gesundheit wäre dies beispielsweise für die finanzielle Situation denkbar, die durch unterschiedliche Säulen der Altersvorsorge sowie durch andere wichtige Quellen (z. B. Erspartes) gespeist werden kann [15]. Die Berücksichtigung von verschiedenen Strukturierungsdimensionen der finanziellen Situation erscheint nicht nur für das übergeordnete Verständnis von Ungleichheitslagen in dieser Lebensphase bedeutsam, sondern womöglich auch für die subjektiven Anpassungserfahrungen der Betroffenen.

Abgesehen davon könnten sich künftige Studien den Anpassungserfahrungen von Personen widmen, die, anders als die Teilnehmer:innen der vorliegenden Untersuchung, nicht mit Erreichen ihres regulären Pensionsalters nach Erwerbstätigkeit pensioniert wurden. So könnte etwa auf Personen mit einer Frühpensionierung oder einer Pensionierung aus vorangegangener Arbeitslosigkeit fokussiert werden, um die Bedeutung solcher Pensionierungsbedingungen für die Anpassungserfahrungen der Betroffenen zu explorieren. Quer zu verschiedenen Übergangsformen erscheint nicht zuletzt auch die qualitative Exploration von mittel- und längerfristigen Anpassungserfahrungen über das erste Pensionsjahr hinaus als empfehlenswert.

## Fazit für die Praxis

- Trotz einer hohen Lebenszufriedenheit nach dem ersten Jahr in der Pension kann die erste Zeit in der neuen Lebensphase von der Bewältigung kritischer Ereignisse und von verschiedenen Gewöhnungserfordernissen geprägt sein. Je nach individuellem Belastungsgrad kann sich daraus auch ein Unterstützungsbedarf ergeben.
- Die allgemeine Lebenszufriedenheit ein Jahr nach der Pensionierung steht aus subjektiver Sicht der Betroffenen mit multiplen Faktoren in Verbindung, insbesondere mit der individuellen Ressourcenausstattung. Dies sensibilisiert für die besondere Berücksichtigung ungleicher Lebenslagen in der Konzeption von Unterstützungsprogrammen rund um den Übergang in die Pension.
- Für manche Personen kann der Pensionseintritt zunächst eine Regeneration von arbeitsbezogenen Belastungen und Erschöpfungszuständen bedeuten. Indirekt zeigt sich darin die Notwendigkeit einer kritischen Evaluation von Arbeitsbedingungen älterer Arbeitnehmer:innen.

## Footnotes

1 Eine anonymisierte Version des quantitativen Datensatzes ist über Zenodo zugänglich: 10.5281/zenodo.5718610.
